# Cannabigerol Attenuates Memory Impairments, Neurodegeneration, and Neuroinflammation Caused by Transient Global Cerebral Ischemia in Mice

**DOI:** 10.3390/ijms26168056

**Published:** 2025-08-20

**Authors:** Nathalia Akemi Neves Kohara, José Guilherme Pinhatti Carrasco, Luís Fernando Fernandes Miranda, Pablo Pompeu Quini, Elaine Del Bel Guimarães, Humberto Milani, Rúbia Maria Weffort de Oliveira, Cristiano Correia Bacarin

**Affiliations:** 1 Department of Pharmacology and Therapeutics, State University of Maringá, Av. Colombo, 5790, Maringá 87020-900, PR, Brazil; pg55595@uem.br (N.A.N.K.); ra123551@uem.br (J.G.P.C.); ra123503@uem.br (L.F.F.M.); ra116773@uem.br (P.P.Q.); hmilani@uem.br (H.M.); 2 Department of Basic and Oral Biology, School of Medicine, University of São Paulo, Av. Bandeirantes, Ribeirão Preto 14015-000, SP, Brazil; eadelbel@forp.usp.br

**Keywords:** bilateral common carotid artery occlusion, glial response, cognition, neuroprotection, cannabigerol

## Abstract

Evidence supporting the clinical use of neuroprotective drugs for cerebral ischemia remains limited. Spatial and temporal disorientation, along with cognitive dysfunction, are among the most prominent long-term consequences of hippocampal neurodegeneration following cerebral ischemia. Cannabigerol (CBG), a non-psychotomimetic constituent of *Cannabis sativa*, has demonstrated neuroprotective effects in experimental models of cerebral injury. This study investigated the neuroprotective mechanisms of CBG in mitigating memory impairments caused by transient global cerebral ischemia in C57BL/6 mice using the bilateral common carotid artery occlusion (BCCAO) model. Mice underwent sham or BCCAO surgeries and received intraperitoneal (i.p.) injections of either a vehicle or CBG (1, 5, or 10 mg/Kg), starting 1 h post-surgery and continuing daily for 7 days. Spatial memory performance and depression-like behaviors were assessed using the object location test (OLT) and tail suspension test (TST), respectively. Additional analyses examined neuronal degeneration, neuroinflammation, and neuronal plasticity markers in the hippocampus. CBG attenuated ischemia-induced memory deficits, reduced neuronal loss in the hippocampus, and enhanced neuronal plasticity. These findings suggest that CBG’s neuroprotective effects against BCCAO-induced memory impairments may be mediated by reductions in neuroinflammation and modifications in neuroplasticity within the hippocampus.

## 1. Introduction

Interest in the pharmacotherapeutic potential of *Cannabis* sp. and its constituents has grown significantly in recent years worldwide [1]. Among more than 100 psychoactive compounds isolated from *Cannabis sativa*, cannabigerol (CBG) has stood out as a promising phytocannabinoid, due to its lack of psychotomimetic effects and its demonstrated anti-inflammatory, antioxidant, and potentially neuroprotective properties (for review see [1,2]). In particular, CBG is also highly lipophilic and easily crosses the blood brain barrier (BBB), making it an attractive compound with plausible positive effects in central nervous system (CNS) diseases [3].

The pharmacological effects of CBG have been associated with a reduction in neuroinflammation and oxidative stress. In vitro, CBG defended neuroblastoma spinal cord (NSC)-34 motor neurons from the toxicity induced by lipopolysaccharide (LPS)-stimulated RAW 264.7, reduced apoptosis, and increased anti-apoptosis protein BcL-2 expression. In the same model, CBG pre-treatment reduced the interleukin (IL)-1β, tumor necrosis factor (TNF)-α, interferon (IFN)-γ, and peroxisome proliferator-activated receptor (PPAR)-γ protein levels [4]. CBG also exerted antioxidant effects and reduced apoptosis in rat CTX-TNA2 astrocytes exposed to hydrogen peroxide (H_2_O_2_) and restored the serotonin level depleted by neurotoxic stimuli in isolated cortexes [5]. In an in vivo model of Huntington’s disease, CBG improved motor deficits, conferred neuroprotection, and attenuated microgliosis [6]. Furthermore, a recent study has revealed that CBG promotes gene expression in initiating cytoskeletal remodeling and axon guidance [7]. All these findings indicate that CBG has potential as a neuroprotective agent. However, few studies using in vivo models of CNS injuries have been conducted using CBG.

The bilateral common carotid artery occlusion (BCCAO) is an animal model of transient global cerebral ischemia (TGCI) and has been used to study neuroprotective therapies for cerebral ischemic conditions [8,9,10]. BCCAO triggers multiple and interconnected pathological events, including increased oxidative stress, neuroinflammation, BBB disruption, and white matter (WM) injury [8,9,11]. A few minutes of BCCAO can produce extensive neuronal loss and impact synaptic plasticity in vulnerable areas of the brain such as the hippocampus [8,9,12]. Alongside neurodegenerative processes, however, restorative mechanisms such as reperfusion, vasculogenesis, neurogenesis, and dendritic remodeling may occur to protect the brain tissue and enable recovery [13]. From a functional point of view, BCCAO in mice results in cognitive deficits and increased anxiety- and depression-like behaviors [8,9,14,15,16]. To the best of our knowledge, no study to date has examined the effects of CBG on the cognitive and pathological responses induced by cerebral ischemia, including the BCCAO model.

The present study aimed to investigate the effects of treatment with CBG on the functional and morphological consequences of TGCI using the BCCAO model in mice. The effects of CBG treatment on behavioral performance were first assessed using the object location test (OLT) and the tail suspension test (TST). Subsequently, we evaluated the impact of the CBG on the expression of proteins associated with neuroinflammation, reflected by microglial and astrocytic activation, using ionized calcium-binding adaptor molecule 1 (Iba-1) and glial fibrillary acidic protein (GFAP), respectively. Glial precursor cells were analyzed through the expression of neural/glial antigen 2 (NG2). We also examined the effects of CBG in ischemic mice on markers of neuronal integrity, including neuronal nuclear protein (NeuN) and microtubule-associated protein 2 (MAP-2), as well as markers of neuroplasticity, such as doublecortin (DCX) and brain-derived neurotrophic factor (BDNF).

## 2. Results

### 2.1. CBG Improves Memory Impairment Induced by BCCAO in Mice

**Figure 1:**
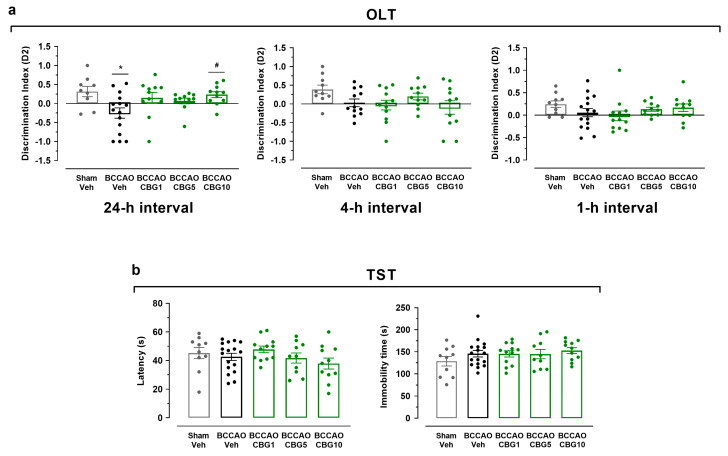
CBG improves memory impairment induced by BCCAO in mice. (**a**) Spatial learning and memory performance was analyzed in the OLT using the D2 exploration index at 1, 4, and 24 h intervals. (**b**) Depression-like behavior was evaluated in the TST by measuring the latency for the first episode of immobility (latency) and the immobility time. The bars represent the mean ± SEM in the different groups (n = 11–17/group). * *p* < 0.05 for sham vs. BCCAO; # *p* < 0.05 for BCCAO vs. BCCAO + CBG (one-way ANOVA followed by Sidak’s test).

Figure 1 show the effects of BCCAO on the behavior of animals subjected to OLT and TST. In the OLT (Figure 1a), a significant main effect among the experimental groups was found at the 24 h interval (F_4,53_ = 3.50; *p* = 0.013) but not at 1 h and 4 h (F_4,53–57_ = 0.90–1.95; *p* > 0.05) intervals. At the 24 h interval, the BCCAO + vehicle (Veh) group presented the lowest D2 scores compared with the sham + Veh group (*p* = 0.016), indicating impairment in long-term spatial memory. This memory impairment was significantly inhibited by CBG at a dose of 10 mg/Kg (CBG10) in comparation with the BCCAO + Veh (*p* = 0.038). When the D2 index in the 24 h interval was analyzed within the group, it was demonstrated to differentiate from zero in the sham + Veh and BCCAO + CBG10 groups (t = 2.32–2.99; *p* = 0.013–0.048), indicating that spatial memory was recovered by CBG10 to the level of the sham group. The same did not occur for the groups with BCCAO treated with the Veh, CBG at a dose of 1 mg/Kg (CBG1), or CBG at a dose of 5 mg/Kg (CBG5) (t = 0.90–1.85; *p* = 0.084–0.380). In the TST (Figure 1b), no between-group differences were detected in the latency for the first episode of immobility (F_4,55_ = 1.332; *p* = 0.27) and the immobility time (F_4,55_ = 0.943; *p* = 0.446).

### 2.2. CBG Decreases Hippocampal Neurodegeneration Induced by BCCAO

**Figure 2:**
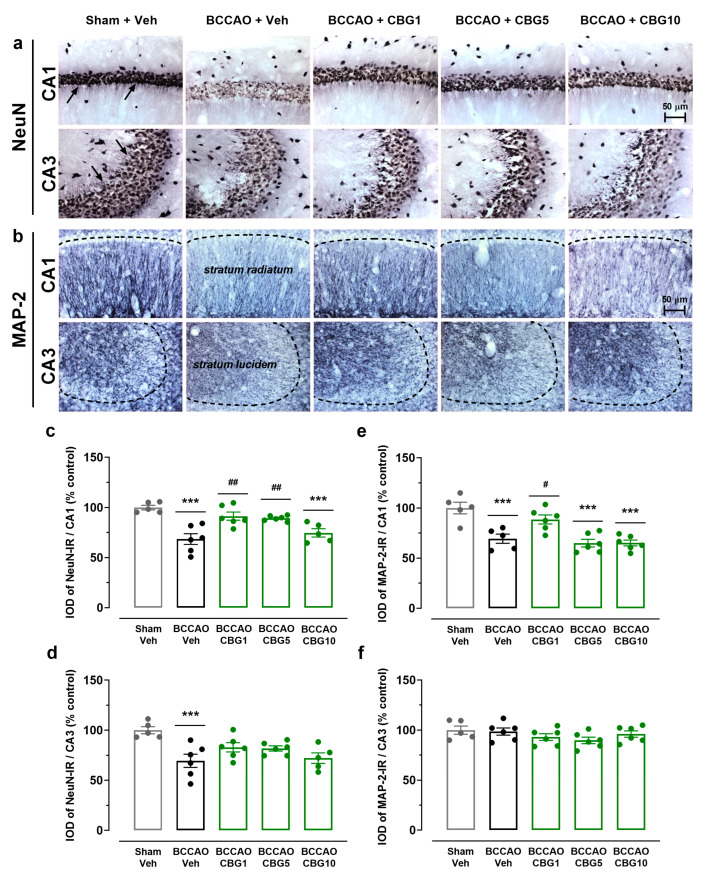
CBG decreases hippocampal neurodegeneration induced by BCCAO. (**a**) Representative photomicrographs of the NeuN-immunoreactive (NeuN-IR) neurons in the CA1 and CA3 hippocampal subfields, indicating intact-appearing neurons (arrows). (**b**) Representative photomicrographs of the MAP-2-immunoreactive (MAP-2-IR) cells in the *stratum radiatum* (CA1 subfield) and *stratum lucidem* (CA3 subfield). The dashed lines delimit the layers of neuronal bodies. (**c**,**d**) Integrated optical density (IOD) of the NeuN-IR neurons in the CA1 and CA3 subfields of the hippocampus. (**e**,**f**) IOD of the MAP-2-IR in the CA1/*stratum radiatum* and CA3/*stratum lucidem*. The bars represent the mean ± SEM in the different groups (n = 5–6/group). *** *p* < 0.001 for sham vs. BCCAO; # *p* < 0.05, ## *p* < 0.01 for BCCAO vs. BCCAO + CBG (one-way ANOVA followed by Sidak’s test).

Hippocampal damage was evaluated by analyzing the expression of NeuN (neuronal body) and MAP-2 (dendrite) in the CA1 and CA3 subfields 14 days after sham or BCCAO surgery (Figure 2). A main effect among the experimental groups was found for the expression of NeuN in the CA1 and CA3 subfields (F_4,23_ = 5.86–11.18; *p* < 0.0001–0.01), indicating the loss of adult pyramidal neurons (Figure 2c,d). Compared to the sham group, these neurons were significantly reduced in the CA1 and CA3 subfields of the ischemic, Veh-treated group (*p* < 0.001). CBG1 and CBG5 decreased this neurodegenerative effect of BCCAO in the CA1 subfield (*p* < 0.01). In the CA3 subfield, however, the neuronal rescue by CBG did not reach statistical significance at the 5% level (*p* > 0.05).

As shown in Figure 2e,f, a main effect among the experimental groups for the expression of MAP-2 was found in the *stratum radiatum* of the CA1 subfield (F_4,23_ = 13.13; *p* < 0.0001), but not in the *stratum lucidem* of the CA3 subfield (F_4,24_ = 1.397; *p* = 0.265). In the *stratum radiatum*, the expression of MAP-2 was significantly reduced in the BCCAO + Veh group compared with the sham + Veh group (*p* = 0.0004), indicating dendritic degeneration. This degeneration and neurohistological sequelae were prevented by CBG1 (*p* = 0.020 vs. Veh). The other two doses had no effect (*p* > 0.05).

### 2.3. CBG Reduces BCCAO-Induced Neuroinflammation

**Figure 3:**
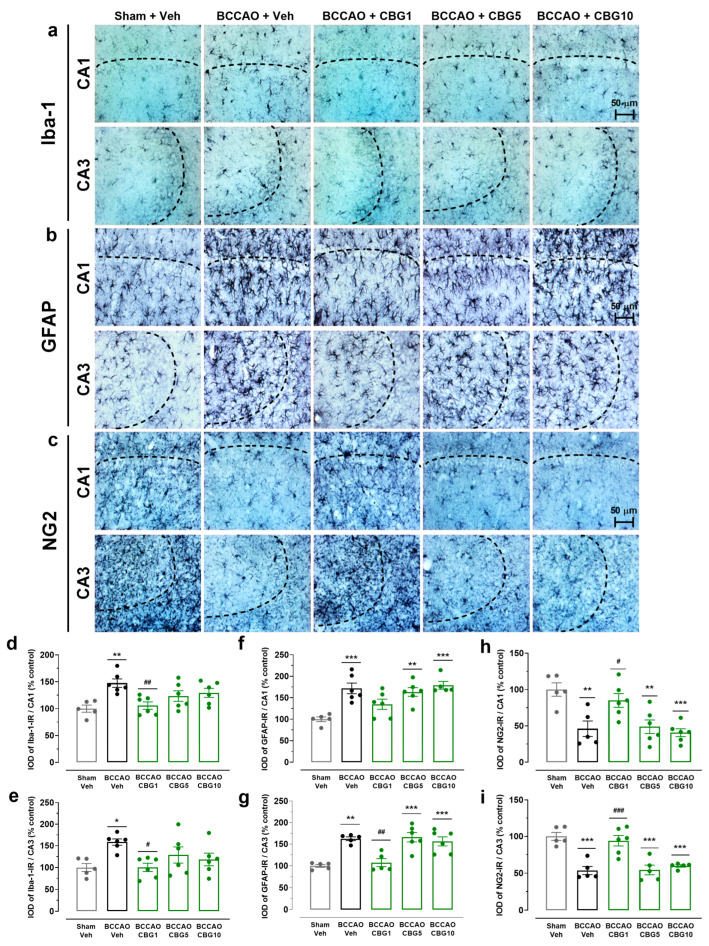
CBG reduces BCCAO-induced neuroinflammation in the hippocampus. (**a**–**c**) Representative photomicrographs of Iba-1-immunoreactive (Iba-1-IR), GFAP-immunoreactive (GFAP-IR), and NG2-immunoreative (NG2-IR) cells in the CA1 and CA3 subfields of the hippocampus. The dashed lines delimit the layers of neuronal bodies. (**d**,**e**) IOD of the microglia (Iba-1-IR) in the CA1 and CA3 subfields of the hippocampus. (**f**,**g**) IOD of the astrocytes (GFAP-IR) in the CA1 and CA3 subfields of the hippocampus. (**h**,**i**) IOD of the glial cell precursor (NG2-IR) in the CA1 and CA3 subfields of the hippocampus. The bars represent the mean ± SEM in the different groups (n = 5–6/group). * *p* < 0.05, ** *p* < 0.01, *** *p* < 0.001 for sham vs. BCCAO; # *p* < 0.05, ## *p* < 0.01, ### *p* < 0.001 BCCAO vs. BCCAO + CBG (one-way ANOVA followed by Sidak’s test).

Neuroinflammation was assessed by analyzing the expression of Iba-1 (microglia), GFAP (astrocytes), and NG2 (glial cell precursor) in the CA1 and CA3 subfields of the hippocampus (Figure 3). The analysis of variance (ANOVA) revealed a main effect among the experimental groups for all these three immunohistochemical markers, in both the CA1 (F_4,23–24_ = 5.38–8.92; *p* < 0.001–0.01) and CA3 (F_4,21–24_ = 3.60–15.56; *p* < 0.0001–0.05). Compared to sham surgery, the BCCAO surgery increased the expression of Iba-1 and GFAP in both the CA1 (*p* < 0.001–0.01; Figure 3d,f) and CA3 subfields (*p* < 0.001–0.05; Figure 3e,g). CBG1 prevented these effects of ischemia in these hippocampal subfields (*p* < 0.01–0.05). In contrast to the expression of Iba-1 and GFAP, the expression of NG2 was reduced in the BCCAO + Veh group, also in the CA1 and CA3 regions (*p* < 0.001–0.01; Figure 3h,i). This effect of ischemia on the expression of NG2 in the CA1 and CA3 hippocampus was abolished by treatment with CBG1 (*p* < 0.001–0.05).

### 2.4. The Impact of CBG on Ischemia-Induced Neuroplasticity

**Figure 4:**
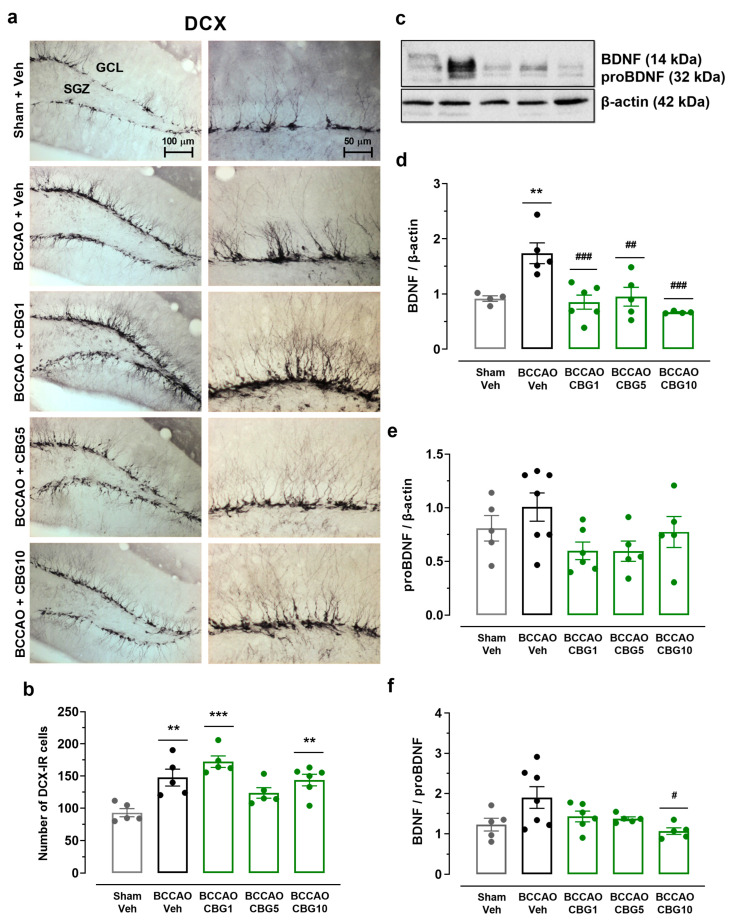
The impact of CBG on ischemia-induced neuroplasticity in the hippocampus. (**a**) Representative photomicrographs of DCX-immunoreactive (DCX-IR) neurons in the GCL and SGZ of the hippocampal dentate gyrus. (**b**) Number of DCX-IR neurons in the SGZ and GCL of the dentate gyrus. (**c**) Representative Western blot bands of BDNF, proBDNF, and β-actin. (**d**,**e**) BDNF and proBDNF protein levels in the hippocampus. (**f**) Ratio between BDNF and proBDNF levels. The bars represent the mean ± SEM in the different groups (n = 5–7/group). ** *p* < 0.01, *** *p* < 0.001 for sham vs. BCCAO; # *p* < 0.05, ## *p* < 0.01, ### *p* < 0.001 for BCCAO vs. BCCAO + CBG (one-way ANOVA followed by Sidak’s test).

Neuronal plasticity in the hippocampus was assessed based on the quantity of newly generated neurons expressing DCX and the concentrations of BDNF and its precursor, proBDNF (Figure 4). The ANOVA revealed a main effect among the experimental groups on the number of DCX-positive neurons within the subgranular zone (SGZ) and granular cell layer (GCL) regions of the dentate gyrus (F_4,21_ = 9.67; *p* < 0.001) (Figure 4b). BCCAO increased the number of DCX neurons (*p* < 0.01 vs. sham), indicating a compensatory response to ischemia. This effect was not altered by CBG (*p* > 0.05 vs. Veh). A main effect among the experimental groups was also observed for BDNF (F_4,19_ = 8.614; *p* < 0.001), but not for the proBDNF levels (F_4,23_ = 2.31; *p* > 0.05) (Figure 4d,e). In animals subjected to BCCAO + Veh, BDNF levels were increased compared to the sham + Veh group (*p* < 0.01). CBG1, CBG5, and CBG10 prevented the increase in BDNF levels when compared with the BCCAO + Veh group (*p* < 0.01). When considering the ratio between BDNF and proBDNF levels (Figure 4f), there was a significant difference among groups (F_4,23_ = 3.274; *p* = 0.029). Although there was an increase in the ratio in the BCCAO + Veh group in comparison to the sham + Veh group, this difference did not reach statistical significance (*p* = 0.091). CBG10 reduced the ratio when compared to the BCCAO + Veh group (*p* = 0.022).

## 3. Discussion

This study evaluates the neuroprotective effects of CBG on behavioral, histological, and biochemical outcomes in a murine model of TGCI induced by BCCAO. To our knowledge, this is the first in vivo investigation demonstrating protective properties of CBG in ischemic brain injury. BCCAO caused memory deficits, hippocampal neurodegeneration, and neuroinflammation. As a compensatory response, BCCAO also increased the expression of BDNF and DCX in the hippocampus of ischemic animals. CBG treatment attenuated memory deficits and neuroinflammation in the hippocampus. Additionally, CBG treatment protected neurons against BCCAO-induced neurodegeneration and restored neuroplasticity markers in the hippocampus.

When tested in the OLT, sham-operated mice successfully discriminated novel from familiar locations at 1, 4, and 24 h post-training (D2 index > 0), reflecting intact spatial memory. In contrast, the BCCAO + Veh group exhibited memory impairment (D2 index < 0), reaching statistical significance only at the 24 h interval. These results differ from previous studies, where BCCAO-induced deficits were observed at 1 and 4 h but not at 24 h [12,16,17]. Although BCCAO did not significantly affect memory at earlier intervals in the present study, the D2 index approached zero, suggesting impaired discrimination in ischemic mice. The discrepancy between studies may stem from differences in stress induced by intraperitoneal (i.p.) injections. In previous studies, injections were administered daily from surgery [12] or pre-surgery [16] until 21–27 days post-ischemia, whereas in the present study, injections ceased on day 7, avoiding overlap with behavioral assessments. This procedural variation may account for differences in ischemia-induced memory deficits. CBG10 significantly mitigated BCCAO-induced memory impairment in the OLT at 24 h post-training, whereas lower doses (CBG1 and CBG5) had no effect. This neuroprotective role of CBG has not been previously documented in in vivo models of cerebral ischemia. However, evidence from other neurodegenerative models supports its potential therapeutic effects. In a Huntington’s disease model induced by 3-nitropropionate, CBG promoted motor function improvement and preserved striatal neurons [6]. Additionally, Fleisher-Berkovich et al. (2023) reported that CBG attenuated neurological deficits and neuronal loss in a model of multiple sclerosis [18]. Collectively, these findings suggest that CBG may contribute to functional recovery following brain injury.

BCCAO did not induce significant behavioral changes in mice when assessed in the TST 14 days post-insult. No comparable studies were available, but Hu et al. (2023) reported increased immobility in a BCCAO model followed by 14 days of chronic unpredictable stress [19]. In that study, the immobility time increased significantly in comparison to the sham-operated mice, indicating depressive-like behavior. However, their study lacked a BCCAO-only group for direct comparison.

BCCAO induced significant neuronal loss in the CA1 and CA3 hippocampal subfields, as evidenced by the expression of NeuN. CBG10 attenuated BCCAO-induced memory deficits; however, this effect was not observed at lower doses (CBG1 and CBG5). Interestingly, neuroprotection in CA1 hippocampal neurons was evident at CBG1 and CBG5, but not at the highest dose (CBG10), suggesting a dissociation between cognitive and neuroprotective effects. Establishing a direct relationship between hippocampal damage and cognitive impairment or between neuronal rescue and functional recovery following global ischemia remains challenging [20]. This complexity likely stems from dysfunction and recovery occurring at subcellular, synaptic, or electrophysiological levels rather than solely from morphological changes measurable by cell counts [21]. Furthermore, CBG-mediated neuronal preservation in CA1 was not detected in the CA3 region. The reason for this selective protection in different hippocampal subfields is unknown. In fact, neurons in the CA1 subfield are more vulnerable to the effects of ischemia [22].

Neuroinflammation is a central aspect of brain ischemia injury that includes the glial activation and rapid synthesis of cytokines and chemokines, which are closely associated with ischemia-induced neurodegeneration [8,23,24,25]. Our analysis demonstrated a persistent glial response following BCCAO in mice, as shown by an increase in the expression of Iba-1 and GFAP in the hippocampus 14 days post-BCCAO. CBG1 prevented both astroglial and microglial responses in both CA1 and CA3 hippocampal subfields of BCCAO mice. Since CBG was administered in the first 7 days after BCCAO, the findings indicate that the neuroprotective effect of CBG occurred in the acute/initial phase of ischemia and that it promoted a sustained anti-inflammatory action.

Previous evidence of CBG-mediated neuroprotective and anti-inflammatory effects has been predominantly derived from in vitro studies. These include models such as oxygen–glucose deprivation in BBB cell cultures, which mimic stroke-like conditions [2], lipopolysaccharide-induced neurotoxicity in spinal cord motor neuron-derived neuroblastoma cells, and excitotoxicity assessed using the MTT assay [4]. The anti-inflammatory properties of CBG are thought to be closely associated with its antioxidant activity, which counteracts ischemia-induced oxidative stress [26,27]. Specifically, CBG has been shown to suppress H_2_O_2_-induced oxidative stress in murine macrophages by downregulating oxidative mediators, including inducible nitric oxide synthase (iNOS), nitrotyrosine, and PPAR-γ. It also inhibits the activation of nuclear factor kappa B (NF-κB) and modulates the mitogen-activated protein kinase (MAPK) signaling pathway. Furthermore, CBG enhances the antioxidant defense system by upregulating superoxide dismutase 1 (SOD-1), thereby preventing cell death [28].

NG2 glial cells are known for their ability to proliferate and generate new oligodendrocytes throughout life [29]. While the NG2-positive cells are mainly oligodendrocyte precursors, some studies suggest their potential differentiation into astrocytes or neurons [30,31]. Furthermore, Sugimoto et al. (2014) reported Iba-1 plus NG2-positive cell accumulation at the peri-infarct/core lesion boundary in a rat stroke model, highlighting the diverse roles of NG2-positive cells [32]. We observed a reduction in the expression of NG2 in CA1 and CA3 hippocampal subfields of BCCAO mice 14 days post-ischemia, aligning with studies showing an initial NG2 increase followed by a decline after 2–3 weeks [33,34]. Herein, CBG1 restored the expression of NG2 to levels comparable to the sham group, potentially in association with changes in the expression of GFAP. This finding suggests the recruitment of NG2-positive cells involved in astrocyte and/or oligodendrocyte differentiation. Indeed, a transient subpopulation of NG2 glia exhibiting astrocyte-like properties has been reported following focal cerebral ischemia, resembling cortical astrocytes and potentially contributing to glial scar formation and tissue repair [35]. Whether the anti-inflammatory effects of CBG modulate this process in the BCCAO model remains to be elucidated and warrants further investigation.

Hippocampal neuroplasticity is a fundamental mechanism of learning and memory. For example, following a cerebral ischemic episode, hippocampal neurogenesis occurs to compensate for damaged cells and neural pathways, typically resulting in the emergence of new neurons in the hippocampal dentate gyrus [36]. In line, ischemic mice showed increases in the expression of DCX in the SGZ and GCL of the dentate gyrus 14 days post-BCCAO, suggesting compensatory neurogenesis. This neurogenesis caused by ischemia is maintained in the CBG treatment groups, with no difference compared to the BCCAO + Veh group. BCCAO animals also showed elevated BDNF expression. BDNF is a neurotrophic factor, and studies have demonstrated that its upregulation is linked to enhanced neurogenesis following cerebral ischemia [37]. The expression of BDNF following ischemic events remains controversial. While some studies report a decrease in BDNF levels [8], others demonstrate an increase [38,39]. Studies employing rats and models of focal cerebral ischemia have demonstrated increased BDNF levels in the lesioned hemisphere at 6 h [39], 24 h, and 8 days [38] of reperfusion, suggesting both the acute and sustained upregulation of BDNF following an ischemic event. The elevated BDNF expression observed in our study may be time-dependent and could coincide with the increased expression of DCX, potentially representing a compensatory response to ischemia. CBG treatment at all three doses reduced BDNF expression to baseline levels despite the neurogenesis increase. Further studies are needed to elucidate these inconsistencies.

MAP-2, crucial for dendritic structure and function, is also a marker of neuronal integrity [40,41]. Its reduction in cerebral ischemia indicates neurodegeneration [42], while increased expression weeks later suggests dendritic recovery and neuroplasticity, linked to memory improvement despite neuronal loss [43]. The expression of MAP-2 decreased in the CA1 hippocampal subfield of BCCAO mice, but no changes were detected in MAP-2 in the CA3 subfield at 14 days post-BCCAO. This regional difference in hippocampal MAP-2 expression following cerebral ischemia remains unclear. Nevertheless, a study using a BCCAO model reported a similar MAP-2 pattern, with reductions in MAP-2 in the CA1 subfield, but not in the CA2 and CA3 subfields, 4 days post-ischemia [44]. CBG1 significantly attenuated the loss of MAP-2 in the CA1 hippocampal subfield. Moreover, CBG1 provided qualitative dendritic protection, suggesting CBG may prevent degeneration and/or promote regeneration.

One limitation of this study is the lack of investigation into the pharmacological mechanisms underlying the effects of CBG in mice subjected to BCCAO. Indeed, CBG appears to act through multiple pharmacological targets. CBG may function as a weak or partial agonist of cannabinoid receptors CB1 and CB2 [45], an agonist of PPARɣ receptors [46], and/or an agonist of transient receptor potential (TRP) channels [47]. Additionally, it acts as an antagonist of the serotonin 1A receptor (5-HT_1A_) and an agonist of the α2-adrenergic receptor [48]. The fact that CBG treatment had protective effects lasting 14 days after BCCAO implies a promising therapeutic action of this compound against the long-term consequences of cerebral ischemia. Many studies still need to be performed to better understand the biological properties of CBG on other pathophysiological events of cerebral ischemia, in addition to its mechanisms of action.

## 4. Materials and Methods

### 4.1. Animals

Male C57BL/6 mice 2 to 3 months old (approximately 30 g) were used. The animals were maintained under standard housing conditions with a 12 h light/dark cycle, controlled temperature (22 ± 1 °C), and had free access to water and commercial chow diet (Nutrilab-CR1^®^; Nuvital Nutrients, Curitiba, Brazil). All experimental procedures were conducted under the approval of the Animal Ethics Committee of the State University of Maringá (CEUA Nº 4320080223).

### 4.2. Drugs

CBG (PurMed Global, Delray Beach, FL, USA) was dissolved in 3% Tween 80 (Synth, Diadema, Brazil) and 3% dimethyl sulfoxide (DMSO, Synth, Diadema, Brazil) in sterile saline (Veh). The animals were randomly assigned to receive i.p. injections of Veh, CBG1, CBG5, or CBG10 for 7 days following BCCAO.

### 4.3. Bilateral Common Carotid Arteries Occlusion (BCCAO)

The surgeries were performed as previously reported [8]. Mice were anesthetized with a mixture of isoflurane/oxygen (1.3–1.5% isoflurane in 100% oxygen, Isoforine^®^, Cristália, São Paulo, Brazil) via a universal vaporizer (Oxigel, São Paulo, Brazil). A ventral neck incision was made to expose the common carotid arteries, which were occluded for 20 min with aneurysm clips (ADCA, Barbacena, Brazil). After the occlusion period, the clips were carefully removed, and the arteries were visually inspected for reperfusion. The incision was then closed with sutures. During the first 3 h post-reperfusion, the animals were kept in a heating box (internal temperature of 30 ± 1 °C) to prevent ischemia-induced cerebral hypothermia [49]. The animals were then returned to their cages with free access to water and food. Some mice underwent exposure of the carotid arteries without occlusion and served as non-ischemic controls (sham). All efforts were made to minimize animal suffering.

### 4.4. Experimental Design

**Figure 5:**
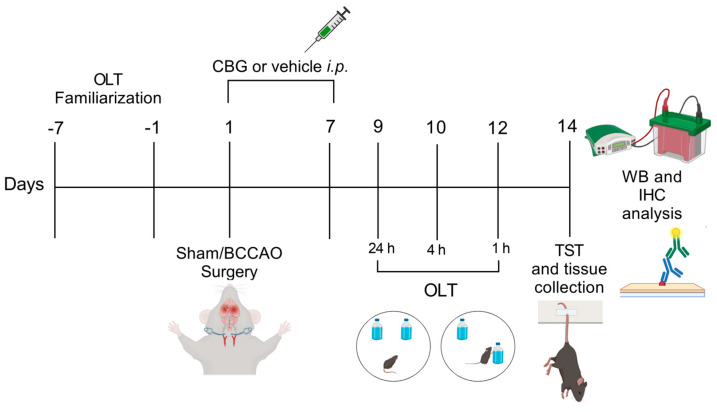
Experimental design. From day −7 to day −1 the animals were familiarized with the OLT environment. On day 1, sham or BCCAO surgery was performed, and Veh, CBG1, CBG5, or CBG10 was administered i.p. for 7 days. Behavioral testing was performed from day 9 to day 14 after BCCAO. Immediately after the last behavioral test, the animals were euthanized, and their brains were processed for immunohistochemical (IHC) and Western blot (WB) analysis. OLT, object location test; TST, tail suspension test.

All the animals were operated on and tested in sequence (Figure 5). C57BL/6 mice were distributed into the following experimental groups: sham + Veh (n = 11), BCCAO + Veh (n = 17), BCCAO + CBG1 (n = 12), BCCAO + CBG5 (n = 11), and BCCAO + CBG10 (n = 11). The pharmacological treatment started 1 h after BCCAO, i.e., at 1 h after reperfusion. Veh or CBG was injected i.p. daily, between 2:00 p.m. and 3:00 p.m., for 7 days. The behavioral tests began 9 days after sham or BCCAO surgery and were conducted during the light phase (between 8:00 a.m. and 1:00 p.m.) in a sound-attenuated experimental room kept at 22 ± 2 °C. The mice were acclimatized to the experimental room for 30 min before each test. On days 9–12, the animals were tested in OLT and on day 14th in the TST. To mitigate potential bias resulting from residual odors left by previous animals, each apparatus was thoroughly cleaned with 70% ethanol followed by water before testing a new mouse. The test sessions were recorded and analyzed using a contrast-sensitive video tracking system (ANYmaze, Stoelting, Wood Dale, IL, USA). After the behavioral evaluation (day 14th), the animals were euthanized with an excessive dose of sodium thiopental i.p. (Thiopentax^®^, Cristalia, São Paulo, Brazil) and had their brains appropriately removed for histological and molecular analysis.

### 4.5. Behavioral Testing

#### 4.5.1. Object Location Test (OLT)

The OLT is designed to evaluate spatial memory and discrimination in rodent models of CNS disorders. This test leverages rodents’ natural tendency to detect changes in the location of objects [50]. It was conducted in an open-field arena where animals were first habituated. The apparatus consisted of a circular arena with a 43 cm diameter and 40 cm high transparent polyvinyl chloride walls. Three distinct sets of objects are used, each one available in triplicate. These objects include (1) an aluminum cube with a tapering top (4.5 cm × 4.5 cm × 8.5 cm), (2) a 200 mL glass bottle filled with water (5.5 cm in diameter, 15.0 cm in height), and (3) a porcelain cube (9.5 cm × 6.5 cm × 6.5 cm). The objects are securely fixed within the arena to prevent movement by the mice. One week before the BCCAO procedure, the animals were introduced to the OLT. Initially, they can explore the empty arena for two consecutive days (3 min per day) to familiarize themselves with the environment. During the next four days, the mice undergo training with the objects until they achieve consistent performance, demonstrating reliable discrimination at a 1 h interval.

Following BCCAO surgery, the OLT was conducted on days 9–12 post-surgery at 24 h, 4 h, and 1 h intervals between the training phase and the test phase. Each test session consists of two trials, each lasting 3 min. In the first trial (T1), two identical objects are placed in the arena, and the mouse was allowed to explore them. After a set time interval (24 h, 4 h, and 1 h), the mouse was returned for a second trial (T2), where one of the objects had been relocated. The amount of time the mouse spent exploring each object during both trials was recorded by an observer that was blinded to the treatment conditions. A discrimination index (D2) was then calculated, reflecting the animal’s ability to perceive the object relocation, i.e., spatial memory. The formula was as follows: D2 = time spent exploring the novel location—time spent exploring the familiar location/total exploration time. This measure corrects for total exploration time during T2, ensuring comparability even if treatment affects overall exploration. Exploration is defined as the mouse directing its nose within 1 cm of the object or touching it with its nose, whereas sitting on the object is not considered exploration.

To reduce potential biases, the order of objects, the identity of the relocated object, and the position of relocation are balanced across the experiment and between groups.

#### 4.5.2. Tail Suspension Test (TST)

The TST was used to assess the increased depression-like behaviors. The animals were positioned in the wall of the box with a white background with adhesive tape. The parameters analyzed were the latency for the first episode of immobility (latency) and the immobility time in the last 4 min of the testing [51]. The camera was positioned in front of the box used to record the animal’s behavior. The test lasted 6 min.

### 4.6. Western Blot

For Western blotting analysis, 5–6 animals from each experimental group were randomly taken and anesthetized with sodium thiopental i.p. (Thiopentax^®^, Cristalia, São Paulo, Brazil). The hippocampus was carefully removed and dissected using spatulas, tweezers, and a table magnifying glass [52]. Each tissue was transferred to an Eppendorf tube containing homogenization buffer (10% glycerol, NaCl; 137 mM, 20 mM Tris HCl, pH 7.5) with protease inhibitor (Sigma-Aldrich, St. Louis, MO, USA). The material was centrifuged in a refrigerated centrifuge at 12,000 rpm for 10 min. Samples were diluted to reach protein concentrations equal to 3 µg/µL. A total of 30–60 μg of proteins were separated by electrophoresis in 12% SDS-polyacrylamide gel. Proteins were transferred to a nitrocellulose membrane and incubated for 45 min in Tris buffer (25 mM Tris base, 192 mM Glycine, 200 mL methanol, pH 8). Blocking was performed with 3% dehydrated milk in tris buffer saline-tween (TBS-T) at room temperature for 1 h. Then, the membranes were incubated with primary antibody in TBS-T (Table 1) overnight at 4 °C. After that, the membranes were incubated for two hours with the horseradish peroxidase (HRP) conjugated rabbit anti-mouse secondary antibody (1:1000, Cat# ab6728, Abcam, MA, USA). All blots were stripped with harsh stripping buffer (20% SDS 10%, 12.5% Tris HCl 0.5 M, and 0.8% β-mercaptoethanol in H_2_O), to assess the protein control β-actin. The revelation was performed with an ECLplus^®^ chemiluminescence kit (Invitrogen, Carlsbad, CA, USA) and the bands were visualized with the aid of Chemi-doc (Bio-Rad Laboratories Inc., Hercules, CA, USA). Specific band intensities were quantified using ImageJ (NIH, Bethesda, MD, USA) and normalized to β-actin protein levels. Results are expressed as the mean ± SEM of the protein level.

### 4.7. Immunohistochemistry

For immunohistochemistry assays, 5–6 animals of each experimental group were deeply anesthetized with sodium thiopental i.p. (Thiopentax^®^; Cristália, São Paulo, Brazil) and transcardially perfused with 0.01 M phosphate-buffered saline (PBS) (2 min; 22 mL/min) followed by 4% paraformaldehyde (PFA) in 0.2 M phosphate buffer (PB) (3 min; 22 mL/min). The brains were post-fixed in the same fixative for 2 h and cryoprotected by immersion in a gradient of sucrose solution in 0.01 M PBS (20% and 30%) for 24 h each at 4 °C. The brains were then quickly frozen in liquid nitrogen and stored at −80 °C. Frozen brains were sectioned coronally at 40 μm using a cryostat (Criocut 1800, Reichert-Jung, Heidelberg, Germany). Thirty-six sections of the hippocampus were collected in six alternating tubes with cryoprotectant solution (15% sucrose and 30% ethylene glycol in PB) and stored at −24 °C until further processing. The sections corresponded to the stereotaxic coordinates (−1.70 to −2.46 mm from Bregma), according to the atlas of Paxinos and Franklin (2004) [53].

Sections were washed 3 times in PBS to remove cryoprotectant. Endogenous peroxidase activity was blocked in 1% H_2_O_2_ (Merck, Darmstadt, Germany) in PBS for 30 min. After washing in PBS with 0.3% Triton X-100 (PBST), sections were incubated with 2% bovine serum albumin (BSA; Sigma-Aldrich, Darmstadt, Germany) in PBST for 60 min at room temperature. Following 3 PBST washes, sections were incubated overnight under constant stirring at 4 °C with primary antibodies (Table 1) overnight. Sections were then incubated with biotinylated anti-rabbit secondary antibody (1:500, Cat# sc-2491, Santa Cruz Biotechnology, Santa Cruz, CA, USA) for 2 h, followed by avidin–biotin complex solution (ABC Kit, Vector Laboratories, Newark, CA, USA) for 2 h at room temperature. Peroxidase color reaction was developed using 0.025% solution of 3,3′-diaminobenzidine (DAB; Sigma-Aldrich, Darmstadt, Germany) and 0.05% H_2_O_2_ with NiCl_2_ for enhanced contrast. The sections were properly washed in 0.01 M PBS and mounted on previously gelatinized slides. Dehydration and diaphanization processes with xylene were performed previously to cover the slides with Permount^®^ (Fisher Scientific, Fair Lawn, NJ, USA) and coverslips.

Quantitative analysis was performed using a Leica DM 2500 microscope with a Leica DF345 FX camera. The number of DCX-immunoreactive (DCX-IR) neurons was manually quantified in the SGZ and GCL of the dentate gyrus in both the right and left brain hemispheres. The results are expressed as the mean ± SEM of 3–4 sections per animal. For NeuN-immunoreactive (NeuN-IR), MAP-2-immunoreactive (MAP-2-IR), Iba-1-immunoreactive (Iba-1-IR), GFAP-immunoreactive (GFAP-IR), and NG2-immunoreactive (NG2-IR) cells, digital microscopic areas were captured bilaterally for the CA1 (0.12 mm^2^) and CA3 (0.15 mm^2^) hippocampal subfields in 3–4 sections per animal. Lighting conditions and magnifications were kept constant to avoid signal saturation. ImageJ software (version 1.54k) was used to obtain IOD [54,55]. The selected images were converted to 16-bit grayscale and the background was subtracted. The threshold for a positive signal was predefined and the IOD was calculated. IOD values were calculated and presented as mean ± SEM of the IOD/area for each experimental group, expressed as a percentage of the sham group.

### 4.8. Statistical Analysis

Statistical analyses were performed using the GraphPad Prism 8.0.2 software. Both the behavioral, immunohistochemistry, and Western blot data fit the assumptions of normal distribution (D’Agostino and Pearson or Shapiro–Wilk tests) and homoscedasticity (Bartlett’s test). Therefore, the one-way analysis of variance (ANOVA) followed by Sidak’s post hoc test were used for between-group comparisons. Additionally, in the TLO test, the paired one-sample *t*-test was used for within-group evaluation of spatial memory by computing the D2 discrimination index values that differ significantly from 0. Statistical significance was set at *p* < 0.05.

## Figures and Tables

**Table 1:** List of primary antibodies used in immunohistochemistry and Western blot assays.

Antibodies (Dilution)	Company	Code
Rabbit anti-Iba-1 (1:1500)	Wako Chemicals, Richmond, VA, USA	019-19741
Rabbit anti-GFAP (1:2000)	Abcam, Waltham, MA, USA	Ab7260
Rabbit anti-NG2 (1:200)	Merck Millipore, Darmstadt, Germany	AB5320
Rabbit anti-NeuN (1:500)	Abcam, Waltham, MA, USA	Ab177487
Rabbit anti-DCX (1:1000)	Cell Signaling Technology, Boston, MA, USA	4604S
Mouse anti-pBDNF (1:300)	Santa Cruz Biotechnology, Dallas, TX, USA	Sc65514
Rabbit anti-MAP-2 (1:500)	Sigma-Aldrich, Darmstadt, Germany	M3696
Rabbit anti-β-actin (1:5000)	Abcam, Waltham, MA, USA	Ab227387

## Data Availability

Data are contained within the article.

## Abbreviations

The following abbreviations are used in this manuscript:

5-HT_1A_	Serotonin 1A receptor
ANOVA	Analysis of variance
BBB	Blood brain barrier
BDNF	Brain-derived neurotrophic factor
BCCAO	Common carotid artery occlusion
BSA	Bovine serum albumin
CNS	Central nervous system
CBG	Cannabigerol
CBG1	CBG at a dose of 1 mg/Kg
CBG5	CBG at a dose of 5 mg/Kg
CBG10	CBG at a dose of 10 mg/Kg
DAB	3,3′-diaminobenzidine
DCX	Doublecortin
DMSO	Dimethyl sulfoxide
GCL	Granular cell layer
GFAP	Glial fibrillary acidic protein
IFN	Interferon
IL	Interleukin
Iba-1	Ionized calcium-binding adaptor molecule 1
iNOS	Inducible nitric oxide synthase
IOD	Integrated optical density
i.p.	Intraperitoneal
IR	Immunoreactive
H_2_O_2_	Hydrogen peroxide
HRP	Horseradish peroxidase
PPAR	Peroxisome proliferator-activated receptor
PBS	Phosphate-buffered saline
PBST	PBS with 0.3% Triton X-100
PB	Phosphate buffer
PFA	Paraformaldehyde
MAP-2	Microtubule-associated protein 2
MAPK	Mitogen-activated protein kinase
NaCl	Sodium chloride
NeuN	Neuronal nuclear protein
NiCl_2_	Nickel chloride
NF-κB	Nuclear factor kappa B
NG2	Neural/glial antigen 2
NSC	Neuroblastoma spinal cord
OLT	Object location test
SGZ	Subgranular zone
SOD-1	Superoxide dismutase 1
TBST-T	Tris buffer saline-tween
TGCI	Transient global cerebral ischemia
TNF	Tumor necrosis factor
TRP	Transient receptor potential
TST	Tail suspension test
Veh	Vehicle
WM	White matter
